# CDNF and MANF regulate ER stress in a tissue-specific manner

**DOI:** 10.1007/s00018-022-04157-w

**Published:** 2022-02-07

**Authors:** Emmi Pakarinen, Päivi Lindholm, Mart Saarma, Maria Lindahl

**Affiliations:** Institute of Biotechnology, HiLIFE Unit, University of Helsinki, 00014 Helsinki, Finland

**Keywords:** CDNF, MANF, ER stress, UPR, Dopamine

## Abstract

Cerebral dopamine neurotrophic factor (CDNF) and mesencephalic astrocyte-derived neurotrophic factor (MANF) display cytoprotective effects in animal models of neurodegenerative diseases. These endoplasmic reticulum (ER)-resident proteins belong to the same protein family and function as ER stress regulators. The relationship between CDNF and MANF function, as well as their capability for functional compensation, is unknown. We aimed to investigate these questions by generating mice lacking both CDNF and MANF. Results showed that CDNF-deficient *Manf*^*−/−*^ mice presented the same phenotypes of growth defect and diabetes as *Manf*^*−/−*^ mice. In the muscle, CDNF deficiency resulted in increased activation of unfolded protein response (UPR), which was aggravated when MANF was ablated. In the brain, the combined loss of CDNF and MANF did not exacerbate UPR activation caused by the loss of MANF alone. Consequently, CDNF and MANF deficiency in the brain did not cause degeneration of dopamine neurons. In conclusion, CDNF and MANF present functional redundancy in the muscle, but not in the other tissues examined here. Thus, they regulate the UPR in a tissue-specific manner.

## Introduction

Genetic compensation refers to changes at the gene expression level that can compensate for the loss of function of another gene. It has been reported to occur in gene knockout (KO) models [1]. For example, proteins with similar structures or functions can compensate for each other. Cerebral dopamine neurotrophic factor (CDNF) and mesencephalic astrocyte-derived neurotrophic factor (MANF) are homologous proteins with a similar two-domain structure distinct from other neurotrophic factors [2]. They have a signal sequence that directs them cotranslationally to the endoplasmic reticulum (ER) and secretory pathway, and an ER retention signal that resembles the canonical KDEL sequence regulating their secretion and retrieval to the ER. The function of MANF—but not CDNF—is shown to be mediated by binding to neuroplastin and sulfatide sulfolipids on the cell surface [3, 4]. CDNF and MANF have cytoprotective effects in different animal disease models, such as Parkinson’s disease, ischemic stroke, and spinocerebellar ataxia [5–9].

Despite the similarities between CDNF and MANF, their respective mouse KO phenotypes differ significantly. *Cdnf*^*−/−*^ mice have functional changes in the midbrain dopamine system and display a reduced number of enteric neurons leading to defects in the gastrointestinal transit [10, 11]. In contrast, *Manf*^*−/−*^ mice in an ICR strain develop severe insulin-dependent diabetes and a drastic growth defect [12]. In the brain, loss of MANF delays migration of cortical neurons, however adult mice do not present functional or morphological changes in the cortex or midbrain dopamine system [13, 14]. Invertebrates such as *Drosophila melanogaster* and *Caenorhabditis elegans* have only one orthologous *Manf/Cdnf* gene, reflecting a possible joint function [3, 15]. The invertebrate homolog is structurally more closely related to MANF than CDNF, which suggests that MANF provides the basic function, while CDNF has evolved in vertebrates to cover other tasks. However, simultaneous loss of CDNF and MANF has not been previously investigated.

ER stress is a cellular condition in which aggregated and unfolded proteins accumulate in the ER lumen. This induces activation of unfolded protein response (UPR), which by various means relieves the stress. In mammalian cells, UPR operates through three branches of signaling cascades induced by ER transmembrane proteins inositol-requiring enzyme 1α (IRE1α), PKR-like ER kinase (PERK) and activating transcription factor 6 (ATF6) [16]. Together these pathways activate adaptive mechanisms by reducing the load of newly synthetized proteins, improving protein folding and by enhancing ER-associated degradation (ERAD). If the UPR is not able to reduce the ER stress, it switches to a terminal UPR that eliminates damaged cells [16]. Under basal conditions, the main ER chaperone, glucose-regulated protein 78 (GRP78; alias BiP) is suggested to bind the luminal domains of all three UPR sensors to prevent their activation. When misfolded proteins accumulate in the ER upon ER stress, GRP78 binds to unfolded proteins and releases the URP sensors leading to IRE1α and PERK dimerization, oligomerization, and auto-transphosphorylation. ATF6 is unmasked from the Golgi localization signals, and cleaved in the Golgi releasing its N-terminal active ATF6 transcription factor. The endoribonuclease activity of IRE1α cytoplasmic domain degrades mRNA and thus, reduces ER protein synthesis load or when chronically activated, leads to inflammasome activation and apoptosis. It also splices *Xbp1* mRNA producing an active transcription factor, sXBP1, which induces gene expression of UPR target genes. The oligomerized IRE1α kinase domain recruits adaptor proteins thereby increasing inflammatory signaling cascades and apoptosis when overactivated. Activated PERK phosphorylates eIF2α, which results in global translational arrest. ATF4 is among proteins that can escape this arrest and mediates expression of various genes, including *Chop*, which induces apoptosis in unresolved ER stress. CDNF and MANF are able to downregulate UPR signaling after ER stress induction in different cell types [2, 17]. All three UPR pathways are downregulated upon CDNF treatment in SOD1 mice modeling amyotrophic lateral sclerosis (ALS) [18]. The loss of endogenous MANF in mice causes increased expression of UPR genes in the pancreas, brain, pituitary gland, and liver [12, 13, 19, 20]. MANF interacts with GRP78 [21], and inhibits its nucleotide exchange [22]. Yet, it is not understood whether CDNF and MANF function in a similar manner as UPR regulators.

Regardless of their similar structure and function related to the UPR regulation, it is not clear whether CDNF and MANF can functionally compensate for each other. Therefore, we generated conventional and conditional CDNF/MANF double KO (dKO) mice. We hypothesized that removal of both factors would aggravate phenotypes seen in individual CDNF or MANF KO mice.

## Materials and methods

### Experimental animals

Mice were housed in a pathogen-free facility with 12 h light and 12 h dark cycle. Mice had *ab libitum* access to food and water, and stayed either in individually ventilated cages or group-housed with their littermates when possible.

Generation of conventional *Cdnf*^*−/−*^ and *Manf*^*−/−*^ mice and their genotyping has been previously described [11, 12]. To generate *Cdnf*^*−/−*^::*Manf*^*−/−*^ mice, we crossed heterozygote mice for the *Cdnf* and *Manf* alleles. For some experiments, such as mRNA analysis of embryonic brain samples, one of the breeding pairs was homozygous for the *Cdnf* allele. This was performed to produce an adequate number of *Cdnf*^*−/−*^::*Manf*^*−/−*^ mice and use littermates for the analysis. Conventional *Cdnf*^*−/−*^::*Manf*^*−/−*^ mice were maintained in an outbred Hsd:ICR background strain.

To generate conditional CDNF/MANF dKO mice, we used *Cdnf*^+/−^ mice and mice with Lox-P sites flanking exon 3 of the *Manf* gene that have been previously described [12]. These floxed *Manf*^*fl/fl*^ mice were crossed with transgenic Nestin-Cre mice, which express *Cre* recombinase under the Nestin promoter [23]. The conditional CDNF/MANF dKO mice were produced by crossing *Cdnf*^+/−^::*Manf*^*fl/*+^::*Nestin*^*Cre/*+^ mice with *Cdnf*^+/−^::*Manf*^*fl/fl*^ mice or *Cdnf*^*−/−*^::*Manf*^*fl/fl*^ mice. Due to the complex breeding scheme, we decided to use *Cdnf*^+/−^ mice as controls, as we have not observed differences between wildtype (WT) and *Cdnf*^+/−^ mice in our previous studies [11]. The conditional CDNF/MANF dKO mice were maintained in a C57BL/6JRccHsd background. As expected, the breeding schemes resulted in unequal group sizes. Behavioural tests were performed blinded.

Samples for histological analysis collected from embryonic day (E) 13.5 animals were post-fixed with 4% paraformaldehyde (PFA). For immunohistochemical analysis, adult mice were first deeply anesthetized with Mebunat, and then transcardially perfused with PBS and 4% PFA. For the analysis of mRNA and protein levels, tissue samples were snap frozen in liquid nitrogen immediately after dissection and stored at −75 °C.

### Enzyme-linked immunosorbent assay (ELISA)

To measure CDNF and MANF protein levels in the mouse cardiac serum, we used our previously designed ELISAs [20, 24]. Mouse MANF (mMANF) ELISA recognized both mouse and human MANF, but it did not recognize human CDNF or respond to serum or tissue lysates derived from MANF KO mice. The sensitivity of mMANF ELISA was 29 pg/ml, and dynamic range from 31.25 to 1000 pg/ml of recombinant mMANF. For mMANF ELISA, serum samples were diluted to 1:40 in blocking buffer (1% casein in PBS-0.05% Tween 20). Recombinant mMANF (CYT-827, ProSpec) was used as a standard. For mouse CDNF (mCDNF) ELISA, serum samples were diluted 1:5 in 3% BSA in PBS. The sensitivity of mCDNF ELISA was 6 pg/ml, and the dynamic range 15.6–500 pg/ml of recombinant mCDNF (5187-CD, R&DSystems). All samples were measured in duplicate. Hemolysis in the samples was detected using absorbance values at 414 nm. Only the samples with Abs_414nm_ < 0.3 indicating low or no hemolysis were used for the analysis to avoid interference with protein concentration values [24].

For the measurement of CDNF and MANF protein in tissue samples, we also used our custom-built ELISAs [20, 24]. Muscle tissues were lysed in a 20 mM Tris buffer (pH 8.0) containing 137 mM NaCl, 2.5 mM EDTA, 1% IGEPAL CA-630, 10% glycerol, 0.5 mM sodium orthovanadate and protease inhibitors (cOmplete, 4693159001, Roche). Tissues were mechanically homogenized in the lysis buffer, incubated on ice, and centrifuged for 20 min at 12,000 rpm at 4 °C. Supernatants were collected. Muscle samples were centrifuged for another 10 min at 13,000 rpm. Total protein concentrations were measured from the samples by DC™ Protein Assay kit I (500–0111, Bio-Rad). For mCDNF ELISA, muscle tissue lysates were diluted 1:2000, pituitary gland lysates 1:20, and pancreas lysates 1:20 in blocking buffer. For mMANF ELISA, muscle tissue lysates were diluted 1:1000, pituitary gland lysates 1:20, and pancreas lysates 1:20,000 in blocking buffer and measured as duplicates. Recombinant human MANF (P-101–100; Icosagen) ranging from 62.5 to 1000 pg/ml was used as a standard. The concentration–response curve of mMANF ELISA was slightly different for recombinant mouse and human MANF proteins, and dilutional linearity of mouse tissue lysates was acceptable only when compared with recombinant human MANF standard [24].

### Blood glucose level measurements

Blood glucose levels were measured from a drop of blood from the tail vein with a glucometer from randomly fed animals [12].

### Analysis of messenger RNA levels

RNA isolation was performed as previously described [13]. Briefly, RNA was first isolated with TRI Reagent (Invitrogen, Thermo Fisher Scientific), then RNA concentrations were measured with NanoDrop. Concentrations were adjusted such that equal amounts of RNA were used for the synthesis of complementary DNA (cDNA). The reaction was catalyzed by Maxima H Minus Reverse Transcriptase (Thermo Fisher Scientific) in the presence of oligo-d(T) (Metabion international) and 10 mM NTP mix (Fermentas UAB). For the cDNA reaction, a sample without the reverse transcriptase was used as a negative control. The mRNA levels of target genes were measured from cDNA samples by real-time quantitative polymerase chain reaction (RT-qPCR). For RT-qPCR, we used SYBR Green master mix (Roche Diagnostics) and Lightcycler® 480 Real-Time PCR System (Roche). Primer sequences are listed in Table 1.

**Table 1:** Primers used in the study

Gene	Forward primer	Reverse primer
*Atf4*	5′–ATG GCC GGC TAT GGA TGA T–3′	5′–CGA AGT CAA ACT CTT TCA GAT CCA TT–3′
*Atf6*	5′–GGA CGA GGT GGT GTC AGA G–3′	5′–GAC AGC TCT TCG CTT TGG AC–3′
*Bcl-2*	5′–AGT ACC TGA ACC GGC ATC TG–3′	5′–GGG GCC ATA TAG TTC CAC AAA–3′
*Bcl-XL*	5′– TGA CCA CCT AGA GCC TTG GA–3′	5′–GCT GCA TTG TTC CCG TAG A–3′
*Chop*	5′–CCA ACA GAG GTC ACA CGC AC–3′	5′–TGA CTG GAA TCT GGA GAG CGA–3′
*Creld2*	5′–CAA CAC GGC CAG GAA GAA TTT–3′	5′–CAT GAT CTC CAG AAG CCG GAT–3′
*Edem1*	5′–AAG CCC TCT GGA ACT TGC G–3′	5′–AAC CCA ATG GCC TGT CTG G–3′
*Erdj4*	5′–TAA AAG CCC TGA TGC TGA AGC–3′	5′–TCC GAC TAT TGG CAT CCG A–3′
*Grp78*	5′–ACC CTT ACT CGG GCC AAA TT–3′	5′–AGA GCG GAA CAG GTC CAT GT–3′
*Grp94*	5′–CGT GTG GAG TAG CAA GAC AGA G–3′	5′–CAT AAG TTC CCA ATC CCA CAC AG–3′
*Pdia6*	5′–TGC CAC CAT GAA TCA GGT TCT–3′	5′– TCG TCC GAC CAC CAT CAT AGT–3′
*sXbp1*	5′–GAG TCC GCA GCA GGT G–3′	5′–GTG TCA GAG TCC ATG GGA–3′
*tXbp1*	5′–CAC CTT CTT GCC TGC TGG AC–3′	5′–GGG AGC CCT CAT ATC CAC AGT–3′
*Txnip*	5′–TCA AGG GCC CCT GGG AAC ATC–3′	5′–GAC ACT GGT GCC ATT AAG TCA G–3′
*Th*	5′–CCC AAG GGC TTC AGA AGA G–3′	5′–GGG CAT CCT CGA TGA GAC T–3′

Primer sequences used for the analysis of mRNA expression

### Western blotting

Brain tissue samples were mechanically homogenized in cold lysis 25 mM Tris–HCl buffer (pH 7.5) composed of 150 mM NaCl, 1% NP-40, 0.5% sodium deoxycholate, 0.1% SDS, protease inhibitors (cOmplete, 4693159001, Roche), and phosphatase inhibitors (PhosStop, 4906837001, Roche). After incubation on ice for 30 min, samples were centrifuged at 13,000 rpm for 20 min at 4 °C. Protein concentrations were measured from the supernatant by a DC™ Protein Assay kit I (500–0111, Bio-Rad). Samples containing equal amounts of proteins were mixed with Laemmli buffer and heated at 95 °C for 5 min. Samples were loaded into 4–15% Mini-Protean TGX Gels (Bio-Rad, 4561083) and after the run, transferred to the nitrocellulose membrane. Membranes were blocked with 5% milk powder in Tris-buffered saline (TBS, pH 7.4) + 0.01% Tween 20 (TBS-T) and incubated with primary antibodies at 4 °C overnight. After washes with TBS-T, a horseradish peroxidase-conjugated secondary antibody was applied for one hour, and signals were detected with enhanced chemiluminescence (Pierce™ ECL Western Blotting substrate, 32106, Pierce, Fisher). Primary antibodies used for Western blotting were the following: anti-GRP78 (1:1000, ab21685, Abcam, RRID:AB_2119834), anti-CHOP (1:1000, sc-757. Santa Cruz Biotechnology, RRID:AB_631365), anti-TH (1:1000, MAB318, Millipore, RRID:AB_2201528), anti-dopamine transporter (DAT) (1:1000, MAB369, Millipore, RRID:AB_2190413), and anti-GAPDH (1:3000, MAB374, Millipore, RRID:AB_2107445). Signal intensities of protein bands on the films were quantified with ImageJ software (Fiji ImageJ 1.52).

Western blotting for the muscle tissue was performed from the samples used for the ELISA, therefore the lysis buffer was different. Otherwise, the protocol was similar to that described above, except the chemiluminescence was detected with LAS-3000 Imager (Fujifilm) or iBright (Thermo Fisher). Intensities of protein bands were quantified with Image Studio Lite software (Version 5.2).

### Open field test

Mice were placed in a corner of 30 × 30 cm open field chambers (Med Associates), and their horizontal and vertical activity was recorded for 30 min as described in [11].

### Immunohistochemical staining

After paraffin removal, 5 µm thick tissue sections were boiled in 10 mM citrate buffer (pH 6.0) for antigen retrieval and washed with TBS. Endogenous peroxidase activity was inactivated by incubation with 0.6% H_2_O_2_ in TBS. After washes with TBS-T, sections were blocked with 1.5% goat serum for one hour. Sections were incubated with primary antibody overnight at 4 °C. The primary antibodies used were anti-insulin (dil 1:1000, A0564, Dako, RRID:AB_10013624), anti-glucagon (dil. 1:500, Dako, RRID:AB_10013726), anti-TH (dil. 1:1000, AB152, Millipore, RRID:AB_390204), anti-GFAP (dil. 1:1000, MAB360, Millipore, RRID:AB_11212597), and anti-IBA1 (dil. 1:1000, ab178846, Abcam, RRID:AB_2636859). Biotinylated secondary antibodies were applied for one hour and the signals from sections were detected with Vector diaminobenzidine peroxidase substrate kit (SK-4100, Vector laboratories). Pancreatic sections were counterstained with hematoxylin after signal detection.

### TH-positive cell counting

Counting of dopamine cell numbers in the substantia nigra was performed by a deep convolutional neural network method provided by Aiforia™ image processing and management platform (Fimmic Oy, Helsinki, Finland). The method has been validated for the purpose of dopamine cell counting [25]. From each mouse, 5 µm thick sections from substantia nigra were stained with a TH antibody. Slides were scanned with the 3DHistech Scanner and uploaded to Aiforia™ image platform. The artificial intelligence model was first trained with the stained sections to recognize TH-positive cell somas, and the proper analysis was performed thereafter. Dopamine cells were counted from five sections within the interval of ~ 70 to 75 µm. Estimation of total dopamine cell number in the substantia nigra was performed by assuming that there were three cell layers between counted sections.

### TH-fiber density measurement

From each mouse, six coronal sections of the striatum within the interval of ~ 100 µm in between were chosen for the analysis. Images of TH-stained sections were obtained with 3DHistec Scanner (Pannoramic P250 Flash II whole slide scanner 3DHistech, Budapest, Hungary). Optical densities of both hemispheres were measured with Image-Pro Analyzer 7.0. Optical density from a cortical region above the corpus callosum was subtracted as a background signal. Data is presented as the optical density per hemisphere.

### Quantification of IBA1 and GFAP stainings

From each mouse, three coronal sections of the striatum within the interval of ~ 200 µm in between were used for the analysis. IBA1- and GFAP-stained sections were scanned with 3DHistec Scanner. Images from the striatum were captured with 10 x magnification from scanned sections. Stained area (pixel value) were quantified using Fiji Image J.

### Statistical analysis

Statistical comparisons between two groups were performed with Student’s unpaired *t*-test when data was normally distributed and variances were similar. One-way analysis of variance (ANOVA) was used to compare three or more groups; significant ANOVA were followed by Tukey’s *post hoc* test. When the *p* value was < 0.05, the difference was considered as statistically significant. The significance of unpaired *t-*test or Tukey’s *post* *hoc* test have been informed in the figures. Statistical analysis was performed with GraphPad Prism 8.0. The data is presented as means; error bars show standard error of the mean (SEM).

## Results

### Decreased CDNF serum levels in Manf^−/−^ mice and increased MANF serum levels in Cdnf^−/−^ mice

**Fig. 1:**
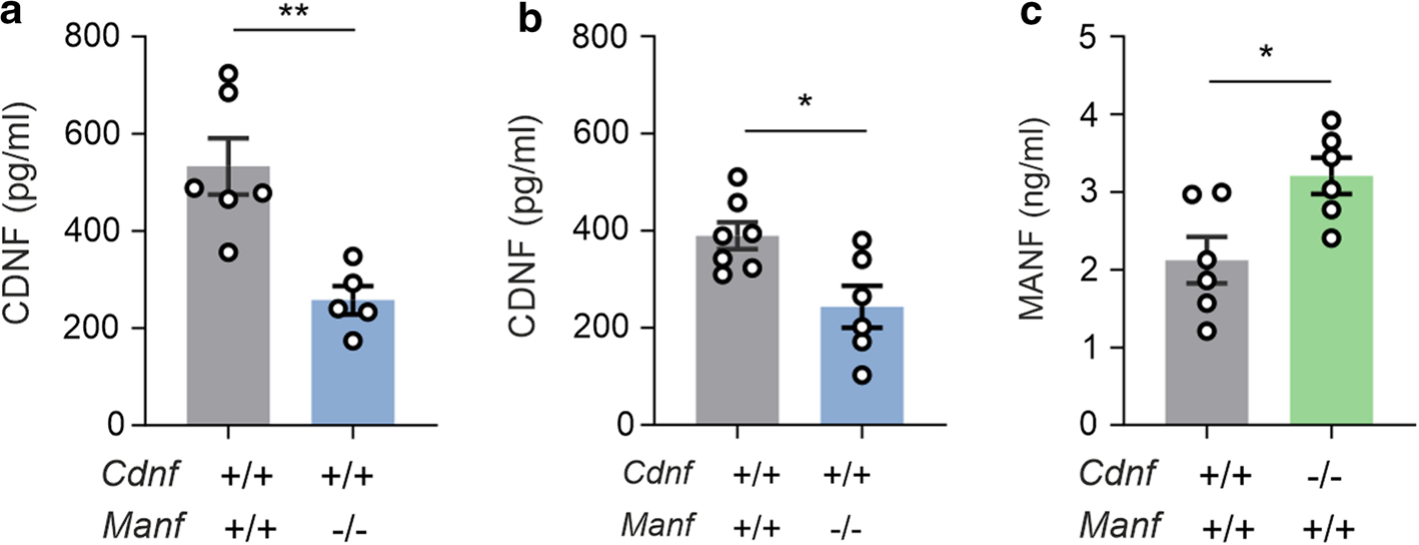
*Manf*^*−/−*^ mice have reduced CDNF serum levels and *Cdnf*^*−/−*^ mice have increased MANF levels. **a** CDNF protein levels determined from cardiac serum of 6–7-week-old *Manf*^+*/*+^ and *Manf*^*−/−*^ littermate male mice by ELISA (*n* = 5–6/genotype). **b** Serum CDNF levels from *Manf*^+*/*+^ and *Manf*^*−/−*^ female mice (*n* = 6–7/genotype). **c** MANF protein levels measured in the sera of 6–7-week-old *Cdnf*^+*/*+^ and *Cdnf*^*−/−*^ littermate female mice (*n* = 6/genotype). The values are reported as mean ± SEM. Unpaired *t*-test was used for the statistical analysis. *Indicates *p* < 0.5 and **p < 0.01

To investigate whether loss of MANF or CDNF in mice alters the levels of the remaining growth factor in the systemic circulation, we analyzed the protein levels of CDNF or MANF in KO mouse sera and compared them to the levels of wildtype (WT) mice. Protein levels in the serum were measured with in-house-developed mouse CDNF and MANF ELISAs from 6−7-week-old mice. Surprisingly, we observed significantly decreased levels of CDNF in *Manf*^*−/−*^ male mice (257.6 ± 29.3 pg/ml) compared with WT male mice (533.1 ± 57.9 pg/ml) (Fig. 1a). Similarly, *Manf*^*−/−*^ female mice (243.7 ± 43.0 pg/ml) had reduced serum CDNF levels compared with WT female mice (389.7 ± 27.8 pg/ml) (Fig. 1b). Furthermore, CDNF protein levels were significantly higher in sera of WT male mice compared to female mice. In contrast, measurements of MANF protein levels from sera of WT and *Cdnf*^*−/−*^ mice showed that female *Cdnf*^*−/−*^ mice had higher MANF protein levels (3.26 ± 0.2 ng/ml) in their sera when compared with female WT mice (2.14 ± 0.3 ng/ml) (Fig. 1c). Preliminary data suggest an increase in MANF levels also in *Cdnf*^*−/−*^ male mice compared to WT male mice (data not shown).

### Removal of CDNF does not change the severity of the Manf^−/−^ mouse phenotype

To study the biological role of CDNF and MANF, as well as the possible compensation between them, we created KO mice lacking both factors. The heterozygous *Cdnf* and *Manf* breedings produced viable *Cdnf*^*−/−*^::*Manf*^*−/−*^ mice. However, at genotyping on postnatal day 21 (P21), the number of *Manf*^*−/−*^, *Cdnf*^+/−^::*Manf*^*−/−*^ and *Cdnf*^*−/−*^::*Manf*^*−/−*^ mice according to the Mendelian distribution was reduced due to an increased perinatal lethality of *Manf*^*−/−*^ mice (Table 2). This supports our previous findings that a significant percentage of *Manf*^*−/−*^ mice in the ICR background die perinatally, although the mechanism behind this has not been studied. However, CDNF deficiency did not increase perinatal lethality in *Cdnf*^+/−^::*Manf*^*−/−*^ or *Cdnf*^*−/−*^::*Manf*^*−/−*^ mice compared to *Manf*^*−/−*^ mice. Actual genotype ratios were measured together from twenty litters, and the distribution of males and females were 47.7% and 52.3%, respectively.

**Table 2:** Genotype ratios at postnatal day 21

Genotype	Expected (%)	Viable P21 mice (%)
*Cdnf* ^+*/*+^::* Manf* ^+*/*+^	6.25	9.13
*Cdnf* ^+*/*+^::* Manf* ^+/−^	12.5	17.83
*Cdnf* ^+*/*+^::* Manf *^*−/−*^	6.25	2.61
*Cdnf* ^+/−^::* Manf* ^+*/*+^	12.5	14.78
*Cdnf* ^+/−^::* Manf* ^+/−^	25	29.13
*Cdnf* ^+/−^::* Manf *^*−/−*^	12.5	5.65
*Cdnf *^*−/−*^::* Manf* ^+*/*+^	6.25	5.65
*Cdnf *^*−/−*^::* Manf* ^+/−^	12.5	12.61
*Cdnf *^*−/−*^::* Manf *^*−/−*^	6.25	2.61

Genotypes of offspring at P21 from crossing *Cdnf*^+/−^::*Manf*^+/−^ mice. Table represents the expected Mendelian ratios and observed ratios. Values have been counted from 20 litters

**Fig. 2:**
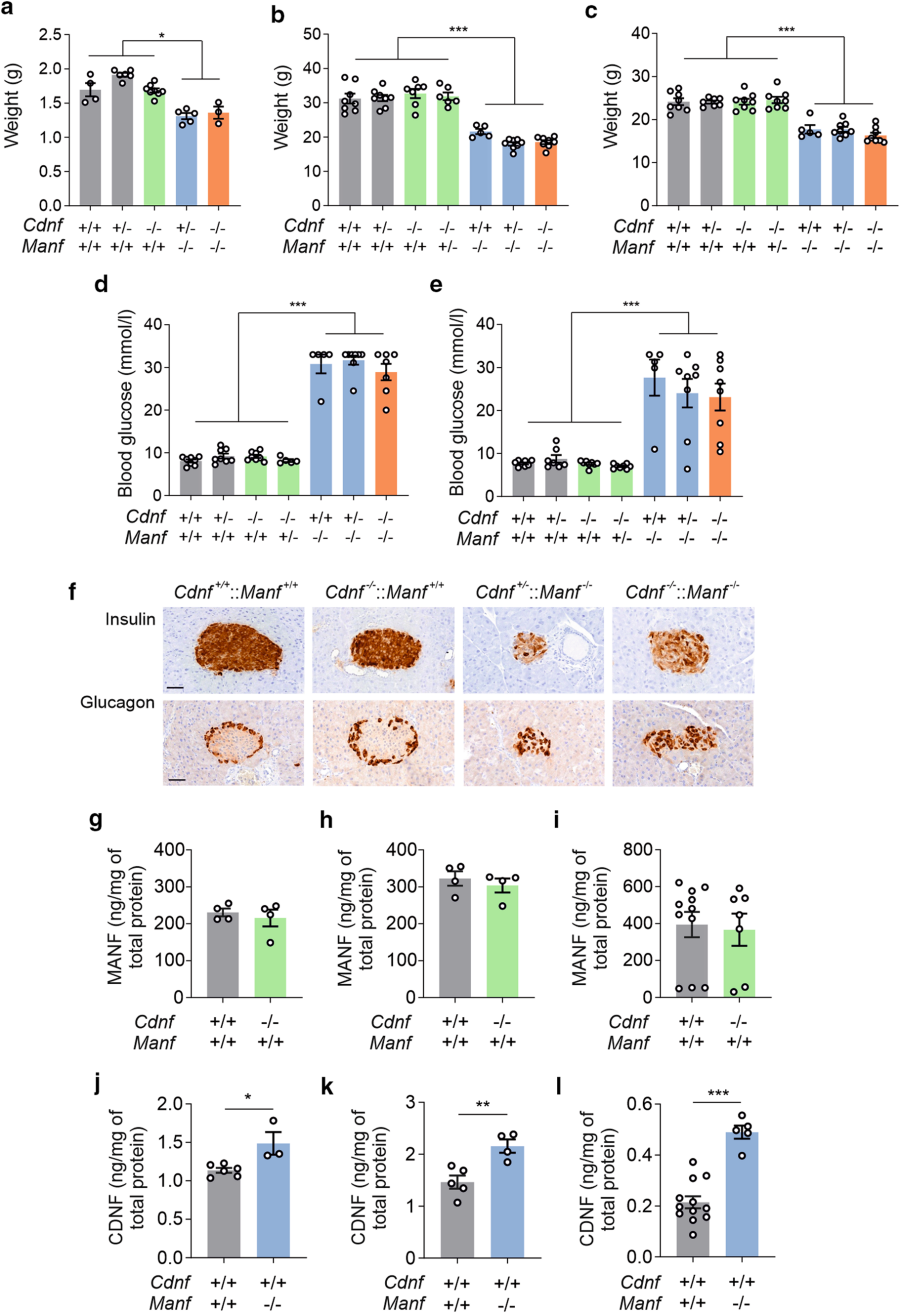
CDNF-deficient *Manf*^*−/−*^ mice have reduced body weights and hyperglycemia similar to *Manf*^*−/−*^ mice. **a** Weights measured from one-day-old mice with indicated genotypes (*n* = 3–8/genotype). **b** Weights of male mice (*n* = 5–8/genotype) and **c** female mice (*n* = 5–8/genotype) with indicated genotypes at the age of 6 weeks, measured between postnatal days (P) 41 to 45. **d** Glucose levels of blood withdrawn from the same male mice (*n* = 5–8/genotype) and **e** female mice (*n* = 5–8/genotype) at the age of P41–45. **f** Pictures of insulin- and glucagon-stained pancreatic islets. Scale bar is 50 µm. **g** MANF protein levels in the pituitary gland of 6-week-old wildtype (WT) and *Cdnf*^*−/−*^ male mice (*n* = 4/genotype) and **h** female mice (*n* = 4/genotype). **i** MANF expression in the pancreas of WT and *Cdnf*^*−/−*^ mice (*n* = 7–11/genotype). **j** CDNF protein levels in the pituitary gland of 6-week-old WT and *Manf*^*−/−*^ male mice (*n* = 3–6/genotype) and **k** female mice (*n* = 4–5/genotype). **l** CDNF protein levels in the pancreas of 6-week-old WT and *Manf*^*−/−*^ male and female mice (*n* = 5–12/genotype).The values are presented as mean ± SEM. One-way ANOVA followed by Tukey’s *post hoc* test and unpaired *t*-test were used for statistical analysis. *p < 0.5, **p < 0.01, ***p < 0.001

We have previously shown that *Manf*^*−/−*^ mice display a severe growth defect already detectable at birth [12]. One of the explaining factors behind the growth defect is a reduced number of growth hormone-producing cells in the pituitary gland and consequently, decreased growth hormone levels [20]. Moreover, MANF ablation has been shown to cause defects in chondrocyte proliferation and bone growth [26]. In contrast, *Cdnf*^*−/−*^ mice develop normally and do not show differences in weight compared to their WT littermates [11]. Since growth defect is one of the most dramatic phenotypes of *Manf*^*−/−*^ mice, and CDNF is expressed in the pituitary gland [20], we measured the weights of mice devoid of both CDNF and MANF. Already at P1, MANF-deficient *Cdnf*^+/−^ and *Cdnf*^*−/*−^ mice had reduced body weights compared to WT mice and mice with CDNF deficiency (Fig. 2a). As *Manf*^*−/−*^ mice have a shorter life span due to progressive overt diabetes and do not survive after two months of age [12], weights were measured at around six week of age, between P41 and P45, when mice were still in visibly good condition. Weights of male (Fig. 2b) and female (Fig. 2c) *Manf*^*−/−*^, *Cdnf*^+/−^::*Manf*^*−/−*^ and *Cdnf*^*−/−*^::*Manf*^*−/−*^ mice were reduced compared with WT, *Cdnf*^+/−^, *Cdnf*^*−/−*^ and *Cdnf*^*−/−*^::*Manf*^+/−^ mice. Thus, the additional CDNF deficiency in *Manf*^*−/−*^ mice does not affect their body weight. Except for the small body size, there were no visible changes observed in CDNF-deficient *Manf*^*−/−*^ mice.

Due to the loss of beta cells in the pancreatic islets, *Manf*^*−/−*^ mice develop insulin-dependent diabetes by the age of P56 [12]. Beta cell death is caused by the prolonged ER stress in the pancreatic islets [12, 27]. Diabetes is the most dominating phenotype of *Manf*^*−/−*^ mice, therefore we investigated the severity of this phenotype in *Cdnf*^*−/−*^::*Manf*^*−/−*^ mice, as CDNF is also expressed in the pancreas [20]. We measured blood glucose levels from random-fed mice of different genotypes and observed that CDNF-deficient *Manf*^*−/−*^ male mice had similarly elevated blood glucose levels as *Manf*^*−/−*^ male mice at P41 − P45 (Fig. 2d). Furthermore, blood glucose levels were higher in all MANF-deficient female mice compared with controls (Fig. 2e). However, there were no differences in the glucose levels between *Manf*^*−/−*^ and *Cdnf*^*−/−*^::*Manf*^*−/−*^ mice. At the time of measurement, blood glucose levels were already high, i.e. on average 30.8 mmol/l in *Manf*^*−/−*^ male mice, 28.9 mmol/l in *Cdnf*^*−/−*^::*Manf*^*−/−*^ male mice, 27.6 mmol/l *Manf*^*−/−*^ female mice, and 23.1 mmol/l in *Cdnf*^*−/−*^::*Manf*^*−/−*^ female mice. To visualize islet morphology in diabetic mice, we stained pancreases from diabetic and control mice with insulin and glucagon antibodies at P41–42. Whereas insulin showed high reactivity in the islets of WT and C*dnf*^*−/−*^ mice, the intensity of insulin staining and number of beta cells were clearly reduced in *Cdnf*^+/−^::*Manf*^*−/−*^ and *Cdnf*^*−/−*^::*Manf*^*−/−*^ mice (Fig. 2f). Thus, the beta cell population in CDNF-deficient *Manf*^*−/−*^ mice was similarly decreased compared to *Cdnf*^+/−^::*Manf*^*−/−*^ mice. The immunostaining with a glucagon antibody revealed intact islets with alpha cells located in the periphery of islets in WT and C*dnf*^*−/−*^ mice, but infiltrated islets with alpha cells revealed disturbed islet morphology in the *Cdnf*^+/−^::*Manf*^*−/−*^ and *Cdnf*^*−/−*^::*Manf*^*−/−*^ mice (Fig. 2f).Thus, the severity of beta cell loss and diabetes in *Manf*^*−/−*^ mice was not affected by CDNF deficiency. Due to the developing severe hyperglycemia in *Manf*^*−/−*^ animals, mice were terminated by the age of 7 weeks and the life span of CDNF-deficient *Manf*^*−/−*^ mice could not be investigated.

Then, we measured MANF and CDNF levels in the pancreases and pituitaries of CDNF- and MANF-deficient mice, respectively. MANF protein levels were analyzed in the pituitary glands of 6-week-old *Cdnf*^*−/−*^ mice by ELISA. Measurements showed that MANF protein expression was not altered in the pituitary glands between WT (230.6 ± 10.7 ng/mg of total protein) and *Cdnf*^*−/−*^ (215.7 ± 22.8 ng/mg of total protein) male mice (Fig. 2g). Similar to male mice, MANF levels were not changed between female WT (322.8 ± 19.6 ng/mg of total protein) and *Cdnf*^*−/−*^ (303.9 ± 18.8 ng/mg of total) mice (Fig. 2h). Interestingly, female WT mice had significantly higher MANF levels in the pituitary glands than male WT mice. Furthermore, MANF levels in the pancreases were measured from random-fed female and male mice and data indicated that values were not significantly different between WT (394.5 ± 68.9 ng/mg of total protein) and *Cdnf*^*−/−*^ mice (366.5 ± 87.7 ng/mg of total protein) (Fig. 2i), although variation was high between samples. Next, CDNF levels were measured from pituitaries of 6-week-old *Manf*^*−/−*^ mice with ELISA. In male *Manf*^*−/−*^ pituitaries, CDNF concentrations were increased (1.49 ± 0.15 ng/mg of total protein) compared to pituitaries in WT mice (1.14 ± 0.03 ng/mg of total protein) (Fig. 2j). CDNF levels were higher in the pituitary glands of female WT mice compared to male WT mice and therefore, groups were not combined. Similar to male mice, CDNF protein expression was higher in the pituitary glands of female *Manf*^*−/−*^ mice (2.16 ± 0.13 ng/mg of total protein) compared to female WT mice (1.46 ± 0.13 ng/mg of total protein) (Fig. 2k). Finally, we measured CDNF protein levels in the pancreases of WT and *Manf*^*−/−*^ male and female mice. Interestingly, CDNF levels were significantly higher in *Manf*^*−/−*^ mice (0.49 ± 0.03 ng/mg of total protein) compared to WT mice (0.21 ± 0.02 ng/mg of total protein) (Fig. 2l).

### GRP78 expression and IRE1α pathway activation are increased in the muscle devoid of CDNF, and the increase is aggravated by MANF ablation

Compared to other tissues, CDNF expression is high in the skeletal muscle while MANF expression is relatively low compared to its expression in other tissues, suggesting that the CDNF has an important role particularly in the skeletal muscle [5, 20, 28]. Therefore, we decided to study UPR activation in the skeletal muscle. The mRNA levels of UPR markers were investigated by RT-qPCR in quadriceps femoris muscles collected from 6-week-old mice.

**Fig. 3:**
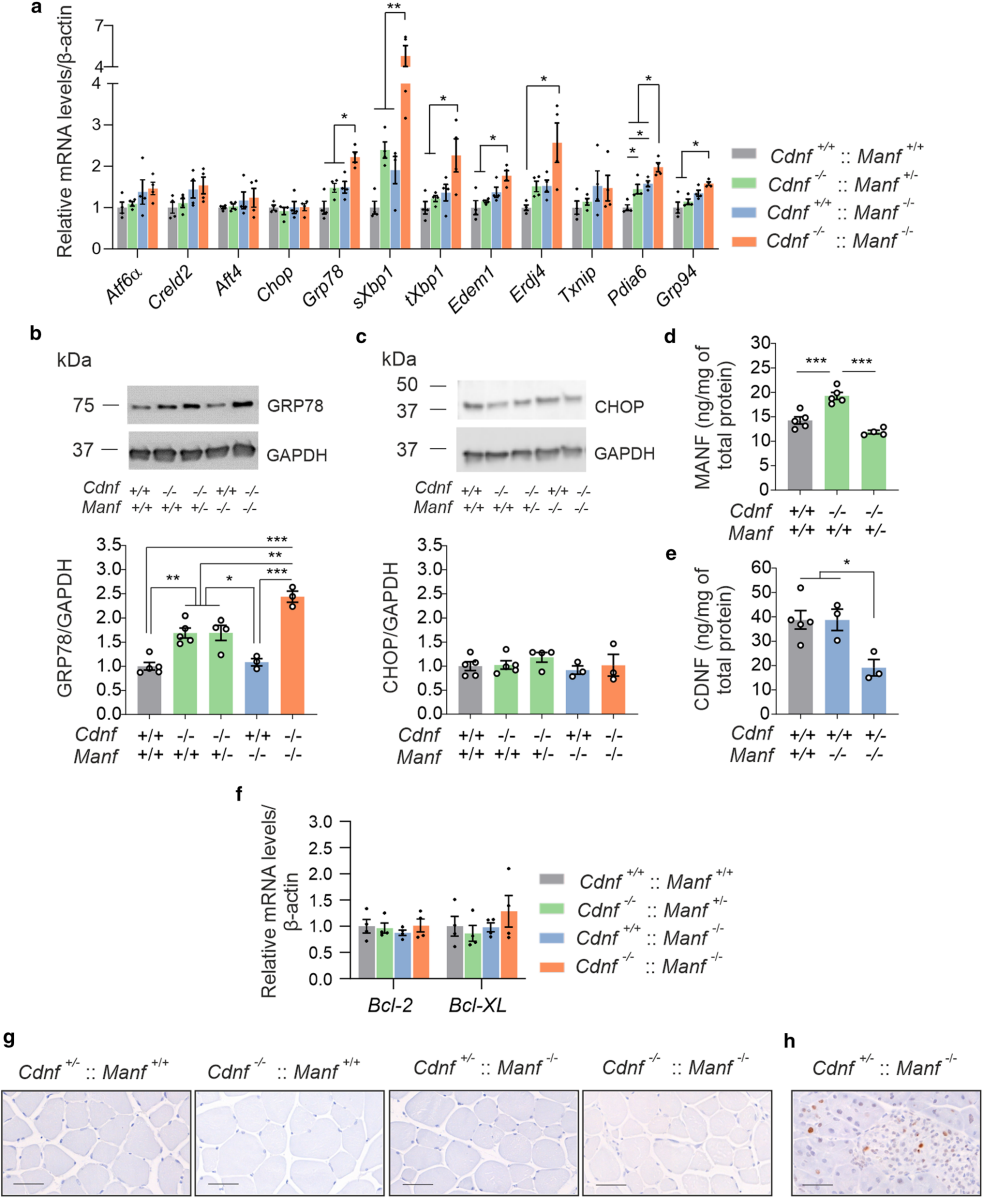
Combined loss of CDNF and MANF aggravates activation of the unfolded protein response in the skeletal muscle. **a** The messenger RNA (mRNA) levels of unfolded protein response (UPR) markers in the quadriceps muscles in 6-week-old male mice (*n* = 4/genotype). **b** Representative images and quantification of Western blots for GRP78 expression and **c** CHOP expression in the quadriceps muscles of female mice (*n* = 3–5/genotype). **d** MANF protein levels in the quadriceps muscle of wildtype (WT), *Cdnf*^*−/−*^ and *Cdnf*^*−/−*^::*Manf*^+/−^ female mice (*n* = 4–5/genotype). **e** CDNF protein levels in the WT, *Manf*^*−/−*^ and *Cdnf*^+/−^::*Manf*^*−/−*^ quadriceps female muscle (*n* = 3–5/genotype) measured by ELISA. **f** The mRNA levels of *Bcl-2* and *Bcl-XL* in the quadriceps muscles of 6-week-old male mice (*n* = 4/genotype). **g** Immunostaining for phosphorylated H2A.X in the quadriceps muscles of 6-week old mice and **h** the pancreas of a *Cdnf*^+/−^::*Manf*^*−/−*^ mouse. Scale bar 50 µm. Data are presented as mean ± SEM. One-way ANOVA followed by Tukey’s *post hoc* test was used for statistical analysis. *p < 0.5, **p < 0.1, and ***p < 0.001

The activated IRE1α ribonuclease domain splices *Xbp1* mRNA to produce a transcription factor (sXBP1*)*, which induces expression of pro-survival genes such as chaperons, genes for lipid synthesis and ERAD components including *Edem1* and *Erdj4* [29, 30]. Interestingly, *Grp78* and IRE1α target genes *sXbp1*, *tXbp1, Erdj4*, and *Edem1* were upregulated in the muscles of *Cdnf*^*−/−*^::*Manf*^*−/−*^ male mice compared with muscles from WT mice (Fig. 3a). Levels of *Grp78*, *sXbp1*, and *Erdj4* were also increased in *Cdnf*^*−/−*^::*Manf*^+/-^ and *Manf*^*−/−*^ mice compared with WT mice, but the increase was not statistically significant. The expression of IRE1α target gene *Txnip* was not increased in *Cdnf*^*−/−*^::*Manf*^*−/−*^ mice, which suggests that the pro-apoptotic arm of the IRE1α pathway was not activated. The levels of *Pdia6*—the binding partner of MANF and regulator of IRE1α [17, 26, 31]—were significantly increased in muscles of *Cdnf*^*−/−*^::*Manf*^+/−^, *Manf*^*−/−*^ and *Cdnf*^*−/−*^::*Manf*^*−/−*^ mice compared with WT mice. Furthermore, the mRNA levels of *Grp94* were increased in *Cdnf*^*−/−*^::*Manf*^*−/−*^ mice compared with WT and *Cdnf*^*−/−*^::*Manf*^+/−^ mice.

In contrast, our results showed that mRNA levels of *Atf4* or pro-apoptotic *Chop* were not increased, indicating that the PERK pathway was unaffected in the muscles devoid of CDNF, MANF or both (Fig. 3a). Similarly, the mRNA levels of *Atf6* and its target gene *Creld2* were not increased (Fig. 3a).

Analyses of the GRP78 protein expression levels from the skeletal muscle with Western blot revealed that the expression was increased in muscles of *Cdnf*^*−/−*^ and *Cdnf*^*−/−*^::*Manf*^+/−^ female mice compared to WT female mice, and that the expression was further increased in mice with the homozygote *Manf* deletion (Fig. 3b). Interestingly, the GRP78 expression levels were similar between WT and *Manf*^*−/−*^ mice. Thus, loss of CDNF leads to increased UPR activation in the muscle, which is further accelerated when MANF is ablated. In addition, we analyzed CHOP protein expression with Western blot from the same samples. Quantification of signal intensities demonstrated that CHOP protein levels were not different between the genotypes analyzed (Fig. 3c). This is in agreement with the data on *Chop* mRNA levels (Fig. 3a).

Since UPR activation was increased in the dKO muscle compared to single KO muscles, we next investigated whether there is possible compensation between CDNF and MANF levels in single KO mouse muscles. First, we measured MANF protein levels in the muscle of *Cdnf*^*−/−*^ female mice by ELISA. Interestingly, MANF levels were increased in *Cdnf*^*−/−*^ muscle (19.3 ± 0.7 ng/mg of total protein) compared with WT muscles (14.3 ± 0.6 ng/mg of total protein), suggesting that the increase in MANF is compensating for the loss of CDNF (Fig. 3d). Accordingly, the muscle samples from *Cdnf*^*−/−*^::*Manf*^+/−^ mice showed only a 17% reduction in MANF expression compared with WT muscle samples. Next, we measured CDNF protein levels in *Manf*^*−/−*^ quadriceps muscle by ELISA. Results showed similar CDNF levels in the muscle of WT mice (38.8 ± 3.8 ng/mg of total protein) and *Manf*^*−/−*^ mice (38.8 ± 4.3 ng/mg of total protein) (Fig. 3e). Muscle samples from *Cdnf*^+/−^::*Manf*^*−/−*^ had exactly half the amount of CDNF expressed in the WT muscle (Fig. 3e). The increase in MANF levels in CDNF-deficient muscle represents a possible compensatory effect of MANF in the CDNF-deficient muscle. On the other hand, loss of MANF in the muscle does not affect the levels of CDNF, suggesting that CDNF is not compensating for MANF function in the muscle.

Finally, we wanted to examine whether there are signs of increased apoptosis in the muscle devoid of CDNF and MANF. We measured mRNA levels of two anti-apoptotic genes, *Bcl-2* and *Bcl-XL*, in the quadriceps muscle of 6-week-old mice but data implicated that there were no changes in the expression of these genes in CDNF-deficient *Manf*^*−/−*^ mice compared with controls (Fig. 3f). In addition, we stained muscle sections for phosphorylated histone variant H2A.X to examine possible chromatin fragmentation and cell death. Stainings did not reveal any cell death in the muscle sections of *Cdnf*^*−/−*^::*Manf*^*−/−*^ mice (Fig. 3g) as compared to the pancreas of *Cdnf*^+/−^::*Manf*^*−/−*^ mice (Fig. 3h).

### CDNF deficiency does not aggravate the intensity of the UPR activation in the brain of Manf^−/−^ mice

We have previously reported that *Manf*^*−/−*^ mice display clearly increased UPR activation in the brain [13]. In that study, we showed that the increase in UPR gene expression was similar in different brain regions of *Manf*^*−/−*^ mice including the cortex, striatum, hippocampus, substantia nigra, and cerebellum. In contrast to *Manf*^*−/−*^ mice, UPR activation is not upregulated in the striatum of adult *Cdnf*^*−/−*^ mouse brain [11]. Here, we wanted to investigate whether the magnitude of UPR activation in the brain is altered in the absence of both MANF and CDNF.

**Fig. 4:**
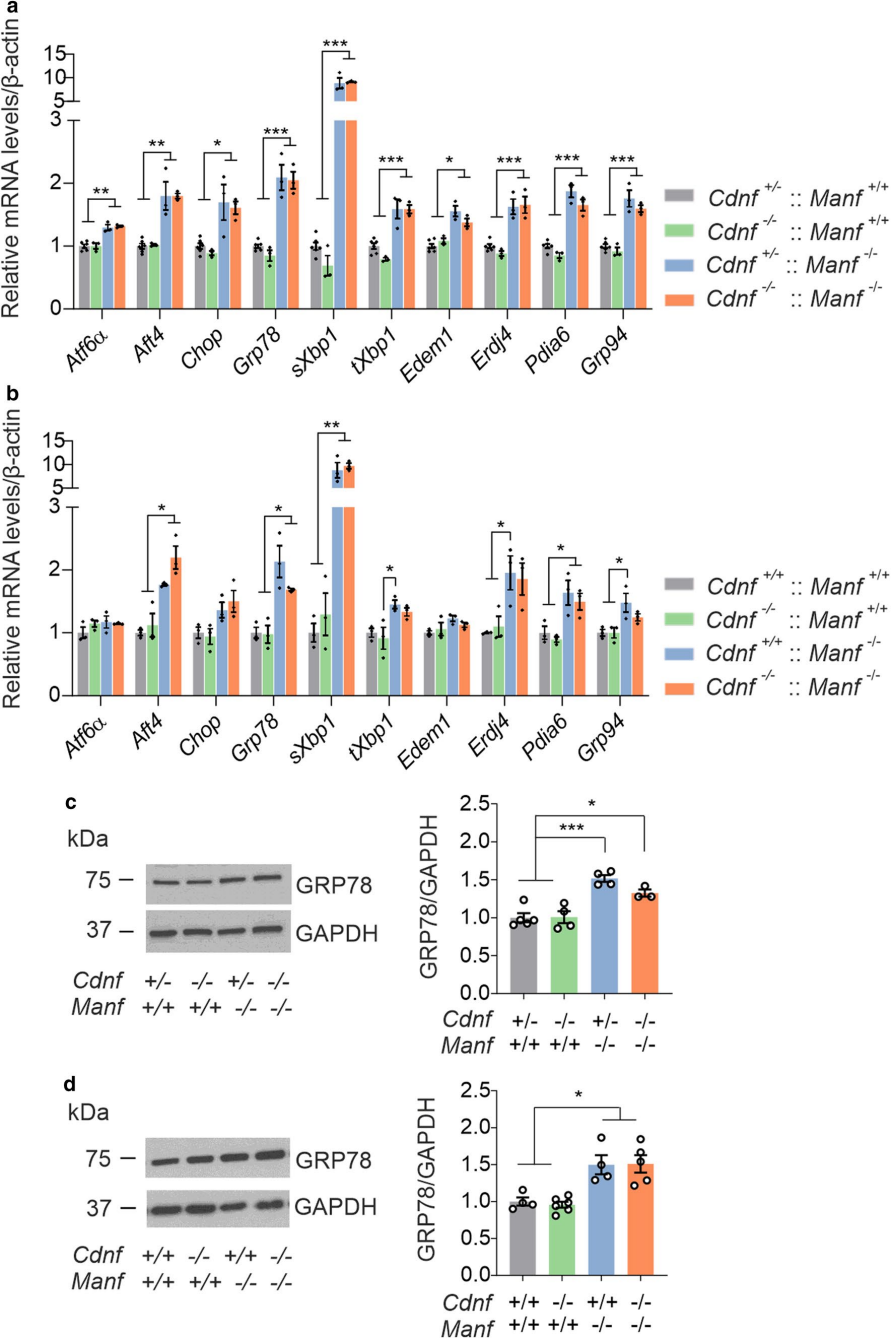
CDNF deficiency does not aggravate activation of the unfolded protein response in the brain of *Manf*^*−/−*^ mice. **a** The messenger RNA (mRNA) levels of the unfolded protein response (UPR) markers in the developing E13.5 mouse brains with indicated genotypes measured by quantitative PCR (*n* = 3–6/genotype). **b** UPR markers in the striatal samples of 6-week-old male mice (*n* = 3/genotype). **c** Representative image and quantification of GRP78 expression on Western blots in postnatal day 1 mouse brains (*n* = 3–5/genotype).** d** Representative Western blot image and quantification of band intensities of GRP78 protein in the striatal samples of 6-week-old male mice (*n* = 4–6/genotype). The values are reported as mean ± SEM. One-way ANOVA followed by Tukey’s *post hoc* test was used for statistical analysis. *p < 0.5, **p < 0.1, and ***p < 0.001

First, we analyzed the UPR activation in the embryonic brain at E13.5 by RT-qPCR since the expression of *Cdnf* mRNA and MANF protein have previously been detected in the mouse brain at this time point [5, 28] and furthermore, *Manf*^*−/−*^ embryos have increased UPR activation already then [13]. We observed that the mRNA levels of *sXbp1* and total *Xbp1* (*tXbp1*) were upregulated in the developing brains of *Cdnf*^+/−^::*Manf*^*−/−*^ and *Cdnf*^*−/−*^::*Manf*^*−/−*^ at E13.5 (Fig. 4a). The mRNA levels of *sXbp1*-induced genes *Edem1* and *Erdj4* were upregulated in *Cdnf*^+/−^::*Manf*^*−/−*^ and *Cdnf*^*−/−*^::*Manf*^*−/−*^ mice. The mRNA levels of *Pdia6* were also elevated in *Cdnf*^*−/−*^::*Manf*^*−/−*^ mice. The PERK downstream components *Atf4* and *Chop* were similarly upregulated, and levels of *Atf6* increased. ATF6 further induces the expression of ER chaperones *Grp78* and *Grp94* [32], which were increased in the developing brains of *Cdnf*^+/−^::*Manf*^*−/−*^ and *Cdnf*^*−/−*^::*Manf*^*−/−*^ mice (Fig. 4a). However, there were no differences in UPR regulation between *Cdnf*^+/−^::*Manf*^*−/−*^ and *Cdnf*^*−/−*^::*Manf*^*−/−*^ mice, indicating that deletion of CDNF does not aggravate UPR activation induced by the lack of MANF in the developing mouse brain.

Next, the mRNA expression levels of the UPR genes were investigated in the brain of the same 6-week-old mice as used for muscle analyses. Striatal samples were dissected from the brains and analyzed by RT-qPCR (Fig. 4b). Results showed that the mRNA levels of *Grp78*, *sXbp1* and *Pdia6* were upregulated in the striata of *Manf*^*−/−*^ mice similarly independent of CDNF deficiency. UPR gene expression in the brain of *Cdnf*^*−/−*^ mice was similar to WT mice. In the PERK pathway, only *Atf4* mRNA levels were increased in *Manf*^*−/−*^ and CDNF-deficient *Manf*^*−/−*^ mice. The mRNA levels of *Atf6* or *Edem1* did not differ in the brain of different genotypes at this age. Furthermore, the mRNA levels of *Erdj4, tXbp1,* and *Grp94* were significantly upregulated in *Manf*^*−/−*^ striata, and there was a clear trend for similar upregulation in *Cdnf*^*−/−*^::*Manf*^*−/−*^ mice. These results are in agreement with our previous data on striata in 5-week-old *Manf*^*−/−*^ mice [13].

We have previously shown that *Grp78* mRNA levels are increased in *Manf*^*−/−*^ brains at P1 [13]. As *Grp78* mRNA levels were changed, we analyzed GRP78 expression at the protein level in the brains of P1 mice by Western blot. Quantification of band intensities revealed a similar increase in GRP78 protein levels in *Manf*^*−/−*^ and *Cdnf*^*−/−*^::*Manf*^*−/−*^ brains (Fig. 4c) as seen for *Grp78* mRNA levels measured with RT-qPCR (Fig. 4b). GRP78 protein expression levels were also examined from striatal samples of 6-week-old mice. Western blot analysis indicated that GRP78 expression levels were increased in *Manf*^*−/−*^ and CDNF-deficient *Manf*^*−/−*^ mice compared to WT and *Cdnf*^*−/−*^ mice (Fig. 4d). However, there were no differences in GRP78 levels between *Manf*^*−/−*^ and CDNF-deficient *Manf*^*−/−*^ mice. Thus, the severity of increased cerebral UPR activation during embryonic development and young adulthood in *Manf*^*−/−*^ mice is not affected by CDNF deficiency.

### Expression of TH and DAT not significantly changed in CDNF-deficient Manf^−/−^ mice

**Fig. 5:**
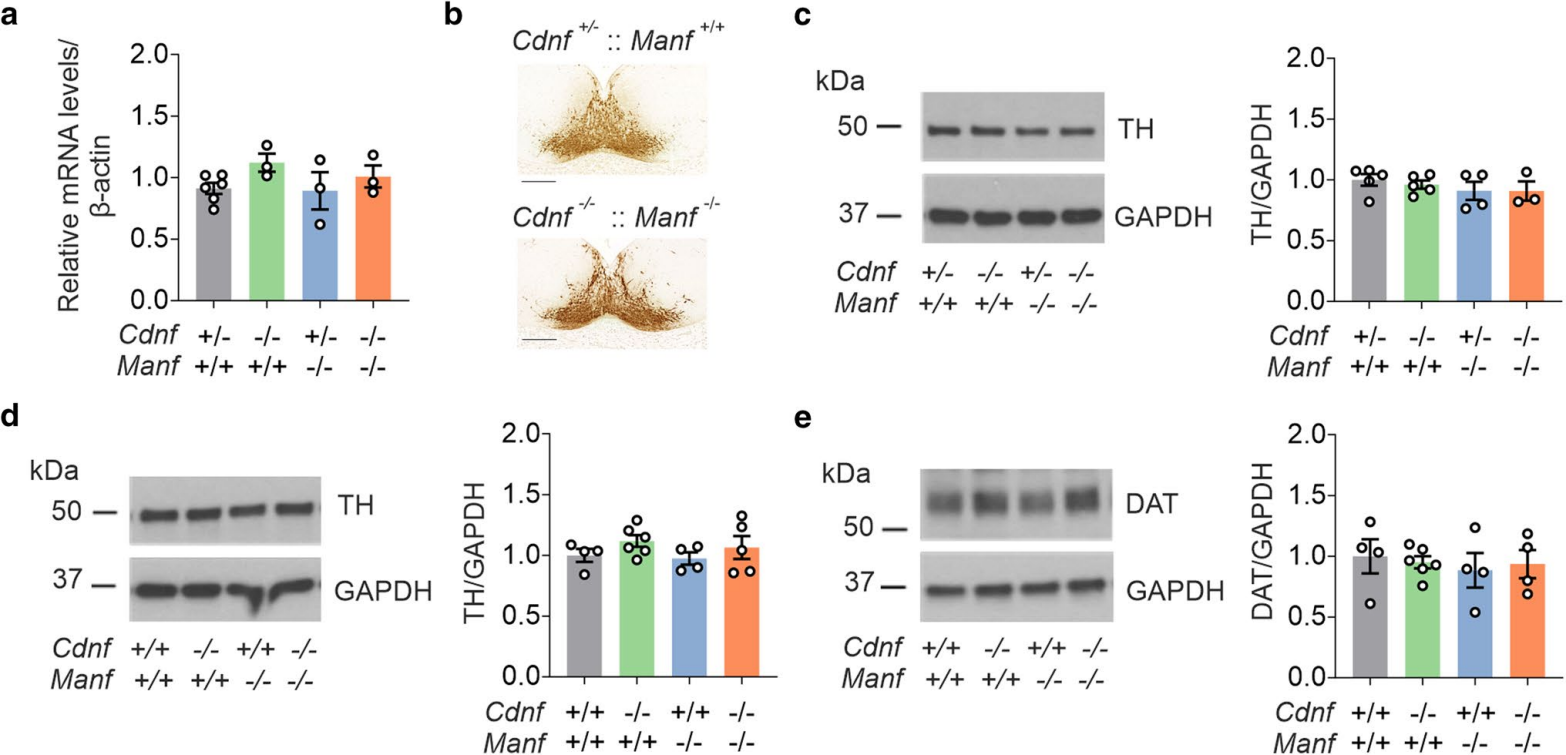
Expression of tyrosine hydroxylase and dopamine transporter is not altered in CDNF-deficient *Manf*^*−/−*^ mice. **a** The messenger RNA levels of tyrosine hydroxylase (*Th)* in the brain of embryonic day 13.5 (E13.5) mice with indicated genotypes (*n* = 3–6/genotype). **b** Images of TH-stained brain midfloor plates in E13.5 *Cdnf*^+/−^::*Manf*^+*/*+^ and *Cdnf*^*−/−*^::*Manf*^*−/−*^ mice. Scale bar is 200 µm. **c** Representative images of Western blot membranes and quantification of TH protein in the brain of postnatal day 1 (P1) mice with indicated genotypes (*n* = 3–5/genotype). **d** Images and quantification of TH protein band intensities of Western blot membranes in samples from striata of 6-week-old male mice with indicated genotypes (*n* = 4–6/genotype).** e** Dopamine transporter **(**DAT) protein expression levels in the striatal samples of 6-week-old male mice (*n* = 4–6/genotype). Data presented as mean ± SEM. One-way ANOVA was used for statistical analysis

As both proteins protect dopamine neurons and are expressed in the midbrain, we investigated the effect of the combined loss of CDNF and MANF in the brain dopaminergic system by analyzing the expression of tyrosine hydroxylase (TH)—the key enzyme catalyzing dopamine synthesis—during brain development and maturation. Neurogenesis of mouse midbrain dopamine neurons takes place between E8.5 and E13.5 [33]. TH expression can be detected at this stage in the developing brain. We measured the mRNA levels of *Th* in the developing brains of *Cdnf*^*−/−*^::*Manf*^*−/−*^ and control mice at E13.5. The RT-qPCR analysis did not show statistical differences between the genotypes (Fig. 5a). In addition, immunostaining for TH protein showed similar staining pattern in the *Cdnf*^+/−^::*Manf*^+*/*+^ and *Cdnf*^*−/−*^::*Manf*^*−/−*^ midbrain floor (Fig. 5b).

Maturation of midbrain dopamine neurons involves two main phases of programmed cell death—at P2 and P14 [34]. Accordingly, we analyzed the TH levels of P1 brains with Western blotting. Protein levels of TH were not altered between *Cdnf*^*−/−*^::*Manf*^*−/−*^ mice and control mice (Fig. 5c). We then analyzed the brain of *Cdnf*^*−/−*^::*Manf*^*−/−*^ mice at the age of 6 weeks. Expression levels of different proteins were measured in striatal samples with Western blotting. Our data indicated similar protein levels of TH in the striatum of CDNF-deficient *Manf*^*−/−*^ mice compared to WT, *Cdnf*^*−/−*^, and *Manf*^*−/−*^ mice (Fig. 5d). Additionally, DAT protein levels were similar between the mice investigated (Fig. 5e).

### CDNF deficiency does not aggravate the midbrain phenotype in aged conditional MANF knockout mice

**Fig. 6:**
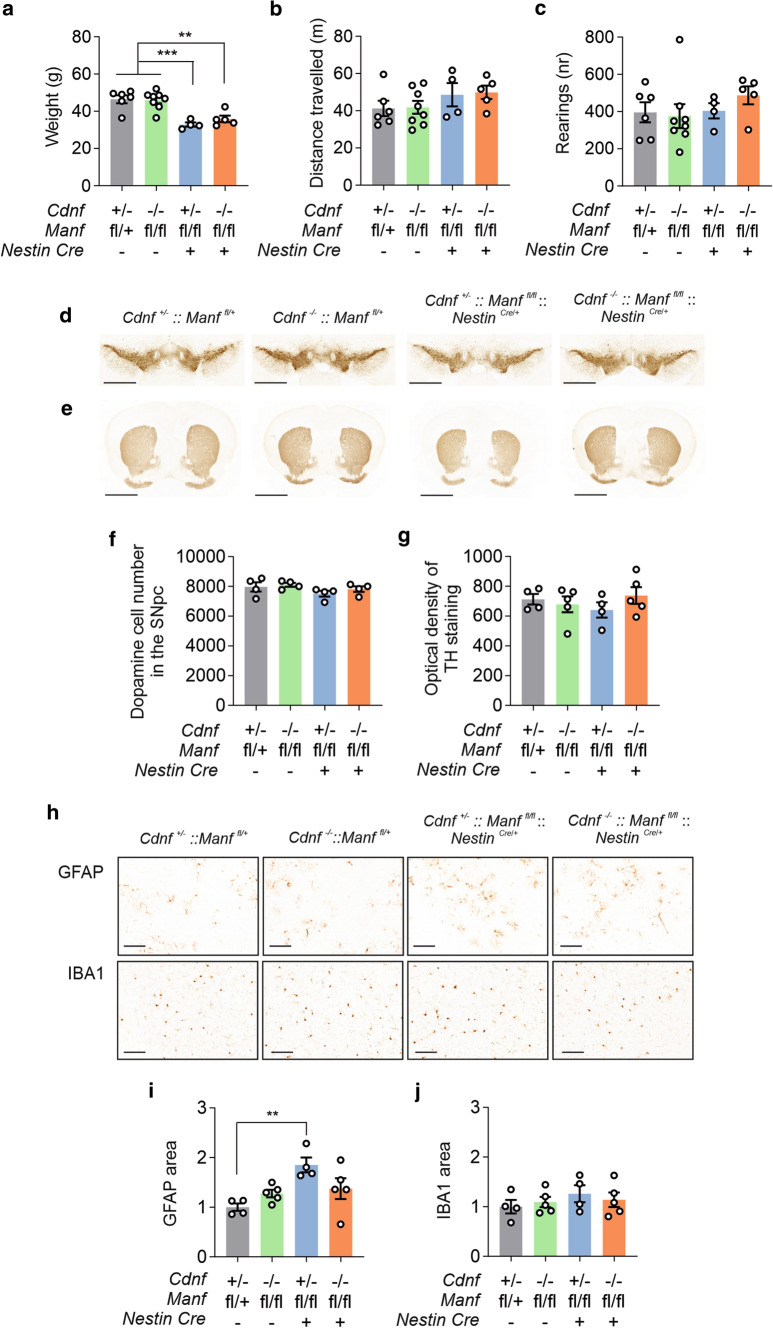
Conditional *Cdnf*^*−/−*^::*Manf*^*fl/fl*^::*Nestin*^*Cre/*+^ mice show no midbrain dopamine neurodegeneration. **a** Weights of 12-month-old male mice with indicated genotypes (*n* = 4–8/genotype). **b** Open field activity of male mice presented as distance travelled and **c** vertical counts (*n* = 4–8/genotype) during 30 min. **d** Representative images of tyrosine hydroxylase (TH)-stained striatal sections and **e** nigral sections of 12-month-old male mice. Scale bars are 2000 µm (d upper row) and 1000 µm (**e** lower row). **f** Total numbers of dopamine neurons in the substantia nigra pars compacta (SNpc) (*n* = 4/genotype). **g** Optical density of TH staining in the dorsal striatum of male mice (*n* = 4–5/genotype).** h** Images of striatal sections immunostained for glial fibrillary acidic protein (GFAP) to detect astrocytes, and ionized calcium binding adaptor protein 1 (IBA1) to detect microglia. Scale bar is 100 µm. **i** Quantification of GFAP-positive area and **j** IBA1-positive area in the striatum of 1-year-old male mice (*n* = 4–5/genotype). The values are presented as mean ± SEM. One-way ANOVA and Tukey’s *post hoc* test were used for statistical analysis. *p < 0.05, **p < 0.01, ***p < 0.001

In mice, MANF and CDNF are expressed in the substantia nigra, where MANF—but not CDNF—has been found to co-localize in TH-positive neurons [5, 20, 28]. We generated conditional neuron-specific CDNF/MANF dKO mice to examine survival and maintenance of neurons during aging. In conditional *Cdnf*^*−/−*^::*Manf*^*fl/fl*^::*Nestin*^*Cre/*+^ mice, CDNF expression was lost from all cells, whereas MANF was specifically removed from neurons and astrocytes. We aged the mice until they were one year old. Measurement of body weights indicated differences in body size between the genotypes. CDNF-deficient *Manf*^*fl/fl*^::*Nestin*^*Cre/*+^ mice and *Manf*^*fl/fl*^::*Nestin*^*Cre/*+^ mice were smaller compared to control mice that were heterozygote or homozygote for *Cdnf* and without the Nestin-Cre transgene (Fig. 6a). This agrees with our previously published data from *Manf*^*fl/fl*^::*Nestin*^*Cre/*+^ mice [13].

Using the conditional CDNF/MANF dKO mice, we addressed the question of whether simultaneous lack of CDNF and MANF in the midbrain would result in the degeneration and death of dopamine neurons. Since locomotor activity is associated with striatal dopamine levels, we first assessed spontaneous locomotor activity of *Cdnf*^*−/−*^::*Manf*^*fl/fl*^::*Nestin*^*Cre/*+^ mice. We have previously shown that the horizontal activity in the open field arena is not impaired in middle-aged *Cdnf*^*−/−*^ or *Manf*^*fl/fl*^::*Nestin*^*Cre/*+^ mice [11, 13]. In agreement with these earlier findings, we showed here that 11-month-old CDNF-deficient *Manf*^*fl/fl*^::*Nestin*^*Cre/*+^ mice had similar behavior in the open field when compared to the three control groups. There were no changes in distance travelled (Fig. 6b) or in the number of vertical counts (Fig. 6c), which both reflect locomotive function. These results imply that the concurrent deficiency of cerebral CDNF and MANF does not reduce locomotor activity in a novel environment.

Next, we analyzed the total amounts of dopamine neurons in the substantia nigra pars compacta (SNpc) and innervation of these neurons to the dorsal striatum. The dopamine neuron number in the SNpc was analyzed on TH-stained sections by a deep-learning algorithm [25]. Results showed no difference in the number of dopamine neurons in the SNpc between CDNF-deficient *Manf*^*fl/fl*^::*Nestin*^*Cre/*+^ mice and control mice (Fig. 6d,f). The innervation of striatal dopamine neuron fibers was investigated by measuring optical density of the TH-stained neurons (Fig. 6e). Quantification of the density of striatal fibers did not show significant differences between the genotypes (Fig. 6g). There was a trend towards lower TH-fiber density in *Cdnf*^+/−^::*Manf*^*fl/fl*^::*Nestin*^*Cre/*+^ mice, but it was not significant (Fig. 6g). The trend was not observed in CDNF-deficient *Manf*^*fl/fl*^::*Nestin*^*Cre/*+^ mice, indicating that combined deficiency of CDNF and MANF does not cause degeneration of dopamine neurons in 1-year-old mice.

We have previously reported that *Manf*^*fl/fl*^::*Nestin*^*Cre/*+^ mice show increased UPR gene expression in various brain regions, including the cortex, hippocampus, midbrain, and cerebellum [13]. As the IRE1α/sXBP1 pathway is involved in inflammatory responses [35], and we have shown that activation of this pathway continues during aging in the substantia nigra of *Manf*^*fl/fl*^::*Nestin*^*Cre/*+^ brains [13], we stained the CDNF-deficient *Manf*^*fl/fl*^::*Nestin*^*Cre/*+^ brains to examine astrocytic and microglial activation. Striatal sections were stained for the astrocyte marker GFAP, and the microglia marker IBA1 (Fig. 6h). Quantification of GFAP-stained sections indicated that GFAP immunoreactivity was significantly increased in *Manf*^*fl/fl*^::*Nestin*^*Cre/*+^ mice compared to control mice (Fig. 6i). However, lack of CDNF did not increase this activation. The analysis of IBA1 stainings, instead, showed that there were not differences in the number of activated microglia between mice analyzed (Fig. 6j).

## Discussion

We present here, for the first time, findings from mice lacking both CDNF and MANF. Our results imply that in general, the phenotype of *Cdnf*^*−/−*^::*Manf*^*−/−*^ mice is highly similar to *Manf*^*−/−*^ mice. We show that global MANF ablation decreases body weight and growth, which is in agreement with previous reports [11, 13]. Our study suggests that CDNF cannot functionally compensate for MANF deficiency in the pituitary gland, as *Cdnf*^*−/−*^::*Manf*^*−/−*^ mice have a similar growth defect as *Manf*^*−/−*^ mice. Measurement of CDNF levels in the pituitary gland of *Manf*^*−/−*^ mice revealed an increase in male and female mice. We may conclude that CDNF cannot functionally compensate for MANF action in the pancreatic beta cells, since at the time of the measurement, blood glucose levels and islet architecture were similar between *Manf*^*−/−*^ mice and CDNF-deficient *Manf*^*−/−*^ mice. Protein levels of CDNF are increased in the pancreas of *Manf*^*−/−*^ mice, but it does not rescue manifestation of diabetes. Given that the CDNF levels in the pancreas are about 1000 times lower than that of MANF, this is not particularly surprising. MANF concentration in the pituitary gland is almost 200 times higher than CDNF concentration, supporting the importance of MANF for the survival of anterior pituitary cells.

The lack of MANF in mice results in a difference in the severity of phenotypes depending on their genetic background. Hearing impairment due to outer hair cell loss was observed in *Manf*^*−/−*^ mice in the ICR background and in the inner ear-specific MANF KO mice in the C57BL/6JRccHsd background, but not in *Manf*^*−/−*^ mice in the CBA strain [36]. Global *Manf*^*−/−*^ mice in the C57BL/6 background are embryonic lethal [26, 37], whereas about 55–60% of the MANF-deficient mice in the ICR background are perinatal lethal (Table 2). A recent study showed that loss of one *Manf* allele in the C57BL/6 background caused an inflammatory and hepatic phenotype in middle-aged mice [38]. We have not detected increased blood sugar levels in aged *Manf*^+/−^ mice in the ICR background (Lindahl, unpublished observations), demonstrating that one *Manf* allele is enough to prevent diabetes manifestation [12]. Interestingly, loss of MANF in humans results in diabetes due to increased ER stress in the pancreatic beta cells analyzed in human stem cell-derived differentiated beta-like cells with a homozygous *MANF* mutation [39]. In addition to diabetes, patients with loss of function mutations in the *MANF* gene showed growth retardation, microcephaly and hearing loss [39, 40]. Our *Manf*^*−/−*^ mice present similar phenotypes to humans, including diabetes, hearing loss and growth defects, indicating that our MANF mouse models are biologically relevant when examining the biological function of MANF in mammals.

We discovered here that CDNF deficiency results in increased expression of UPR genes in the mouse skeletal muscle. This is the first time that loss of endogenous CDNF has been demonstrated to cause ER stress in vivo in mammalian tissue. We observed that CDNF protein levels in the muscle are higher than MANF levels, although generally CDNF protein levels in tissues are about 100 times lower than MANF levels [20]. Simultaneous loss of CDNF and MANF led to even higher expression of UPR genes in the muscle than what was seen in WT and single KO mice, indicating further increased UPR activation in the dKO mice. This indicates that both CDNF and MANF function as ER stress regulators in the skeletal muscle. Given that the IRE1α pathway activation and GRP78 expression are significantly increased, we conclude that adaptive UPR is activated in the muscles devoid of CDNF and MANF. There were no increase in proapoptotic CHOP levels or in the mRNA levels of *Txnip*, *Bcl-*2 or *Bcl-XL*. Thus, terminal UPR is not activated at the time of 6 weeks of age. With only the IRE1α pathway activated, *Manf*^*−/−*^ muscles do not show similar severe ER stress compared to the pancreas and pituitary gland of *Manf*^*−/−*^ mice, where all three conserved UPR pathways are chronically upregulated [12, 20]. Compared to the pancreas, MANF expression in the muscle is much lower, which could partly explain this difference.

Skeletal muscles have a specialized ER called sarcoplasmic reticulum, which has high calcium concentrations, as muscle contraction is based on the release and reuptake of calcium [41]. Recently, exogenous CDNF was demonstrated to restore calcium transients after the treatment with a sarcoendoplasmic calcium ATPase inhibitor, thapsigargin, in human induced pluripotent stem cells differentiated into cardiomyocytes [42]. In the same study, CDNF reduced thapsigargin-induced GRP78 expression in cardiomyocytes. Expression of CDNF is similarly high in the heart as in the skeletal muscle [20]. Both cardiac and skeletal muscles are striated muscles composed of sarcomeres, and their function is dependent on calcium. Therefore, the role of CDNF could be to maintain UPR homeostasis in the calcium-rich environment of skeletal and cardiac muscles.

Compared to muscle, the expression level of CDNF in the brain is modest [20]. We show here that in the developing brain, loss of CDNF does not cause increased expression of UPR genes compared to WT mice. Moreover, loss of CDNF in addition to MANF ablation does not enhance UPR activation that is already present in the MANF-deficient brains. Our data suggests that the role of CDNF in regulating UPR in the brain is less prominent compared to the role of MANF, and that CDNF is not functionally compensating for the loss of MANF in the MANF-deficient brain. The unchanged *Cdnf* mRNA levels in the MANF-deficient brain also supports this suggestion [13].

In rodent Parkinson’s disease models, CDNF and MANF have been shown to promote the survival of dopamine neurons [5, 6, 43–49]. Furthermore, it has been demonstrated that MANF-deficient fruit flies and zebrafish, as well as CDNF-ablated zebrafish have a dopaminergic phenotype [15, 50, 51]. Therefore, CDNF and MANF have been hypothesized to be survival factors for dopamine neurons, which degenerate in Parkinson’s disease and hence lead to the characteristic motor symptoms. However, our previous studies in MANF and CDNF KO mice have shown that endogenous CDNF and MANF are dispensable for dopamine neuron survival [11, 13]. Here, we demonstrate that embryonic removal of both CDNF and MANF in the brain does not result in a degeneration or loss of dopamine neurons in the substantia nigra of aged mice. Due to the small number of mice, we were not able to analyze striatal dopamine levels, which would have complemented the immunohistochemical analysis. However, motor behavior was not altered, which indicates similar dopamine levels in CDNF-deficient *Manf*^*fl/fl*^::*Nestin*^*Cre/*+^ and control mice. We also examined here the early maturation of the midbrain dopamine neurons in mice lacking CDNF, MANF, or both, but did not observe any significant alterations.

As mentioned above, exogenous CDNF and MANF function as survival factors for dopamine neurons in neurotoxin-induced insult models. However, without an insult, exogenous CDNF and MANF are actually not affecting nigrostriatal dopamine neurons in vivo [43, 47]. Similarly, the lack of endogenous CDNF or MANF is not affecting survival or morphology of dopamine neurons in mice [11, 13]. Thus, the survival-promoting function of neuronal CDNF and MANF in vivo is connected to stress conditions. Supporting this idea, *Manf*^*fl/fl*^::*Nestin*^*Cre/*+^ mice were discovered to be more vulnerable to stroke [52], as well as ethanol- and tunicamycin-induced toxicity [53]. Moreover, MANF was shown not to affect naïve dopamine neurons in vitro, but to display an anti-apoptotic activity for ER-stressed neurons, strengthening the idea that additional stress induces MANF to exert its neuroprotective activity [17]. We observed signs of increased GFAP activation in the striata of 1-year-old *Manf*^*fl/fl*^::*Nestin*^*Cre/*+^ mice, which could indicate for a predisposed vulnerability in case of injury.

Our results suggest that there is an interaction between *Cdnf* and *Manf* genes, as we can see increased MANF serum levels in CDNF-deficient mice and vice versa—decreased CDNF serum levels in MANF-deficient mice. The source of MANF and CDNF in the serum is unknown. The finding of clearly reduced CDNF protein levels in *Manf*^*−/−*^ serum can also be a consequence of hyperglycemia and diabetes, although this has not been reported before. The finding of CDNF deficiency affecting MANF serum levels could indicate possible compensation, as a similar increase in MANF expression is also seen in the *Cdnf*^*−/−*^ muscle. On the other hand, sXBP1 has been shown to induce *Manf* mRNA expression [30], and as *sXbp1* mRNA levels are upregulated in the *Cdnf*^*−/−*^ muscle, MANF expression could be a consequence of this. In the brain of *Cdnf*^*−/−*^ mice, neither *sXbp1* levels nor *Manf* mRNA levels are increased [11]. The molecular mechanism behind this compensation requires investigation in future studies.

To summarize, we present here novel findings about conventional and conditional CDNF/MANF dKO mice, and provide new information about the function of endogenous CDNF and MANF. Based on our results, we conclude that CDNF and MANF present redundancy in some—but not all—mouse tissues. Importantly, they both are indispensable for proper UPR regulation, and have specific, different target tissues where they exert their function as ER stress regulators.

## Data Availability

Data and material are available upon request.

## References

[1] El-Brolosy MA, Stainier DYR (2017). Genetic compensation: a phenomenon in search of mechanisms. PLoS Genet.

[2] Lindahl M, Saarma M, Lindholm P (2017). Unconventional neurotrophic factors CDNF and MANF: structure, physiological functions and therapeutic potential. Neurobiol Dis.

[3] Bai M, Vozdek R, Hnízda A, Jiang C, Wang B, Kuchar L, Li T, Zhang Y, Wood C, Feng L, Dang Y, Ma DK (2018). Conserved roles of C. elegans and human MANFs in sulfatide binding and cytoprotection. Nat Commun.

[4] Yagi T, Asada R, Kanekura K, Eesmaa A, Lindahl M, Saarma M, Urano F (2020). Neuroplastin modulates anti-inflammatory effects of MANF. iScience.

[5] Lindholm P, Voutilainen MH, Laurén J, Peränen J, Leppänen VM, Andressoo JO, Lindahl M, Janhunen S, Kalkinen N, Timmusk T, Tuominen RK, Saarma M (2007). Novel neurotrophic factor CDNF protects and rescues midbrain dopamine neurons in vivo. Nature.

[6] Voutilainen MH, Bäck S, Pörsti E, Toppinen L, Lindgren L, Lindholm P, Peränen J, Saarma M, Tuominen RK (2009). Mesencephalic astrocyte-derived neurotrophic factor is neurorestorative in rat model of Parkinson's disease. J Neurosci.

[7] Tadimalla A, Belmont PJ, Thuerauf DJ, Glassy MS, Martindale JJ, Gude N, Sussman MA, Glembotski CC (2008). Mesencephalic astrocyte-derived neurotrophic factor is an ischemia-inducible secreted endoplasmic reticulum stress response protein in the heart. Circ Res.

[8] Zhang GL, Wang LH, Liu XY, Zhang YX, Hu MY, Liu L, Fang YY, Mu Y, Zhao Y, Huang SH, Liu T, Wang XJ (2018). Cerebral dopamine neurotrophic factor (CDNF) has neuroprotective effects against cerebral ischemia that may occur through the endoplasmic reticulum stress pathway. Int J Mol Sci.

[9] Yang S, Huang S, Gaertig MA, Li XJ, Li S (2014). Age-dependent decrease in chaperone activity impairs MANF expression, leading to Purkinje cell degeneration in inducible SCA17 mice. Neuron.

[10] Chalazonitis A, Li Z, Pham TD, Chen J, Rao M, Lindholm P, Saarma M, Lindahl M, Gershon MD (2020). Cerebral dopamine neurotrophic factor is essential for enteric neuronal development, maintenance, and regulation of gastrointestinal transit. J Comp Neurol.

[11] Lindahl M, Chalazonitis A, Palm E, Pakarinen E, Danilova T, Pham TD, Setlik W, Rao M, Võikar V, Huotari J, Kopra J, Andressoo JO, Piepponen PT, Airavaara M, Panhelainen A, Gershon MD, Saarma M (2020). Cerebral dopamine neurotrophic factor-deficiency leads to degeneration of enteric neurons and altered brain dopamine neuronal function in mice. Neurobiol Dis.

[12] Lindahl M, Danilova T, Palm E, Lindholm P, Võikar V, Hakonen E, Ustinov J, Andressoo JO, Harvey BK, Otonkoski T, Rossi J, Saarma M (2014). MANF is indispensable for the proliferation and survival of pancreatic β cells. Cell Rep.

[13] Pakarinen E, Danilova T, Võikar V, Chmielarz P, Piepponen P, Airavaara M, Saarma M, Lindahl M (2020). MANF ablation causes prolonged activation of the UPR without neurodegeneration in the mouse midbrain dopamine system. eNeuro.

[14] Tseng KY, Danilova T, Domanskyi A, Saarma M, Lindahl M, Airavaara M (2017). MANF is essential for neurite extension and neuronal migration in the developing cortex. eNeuro.

[15] Palgi M, Lindström R, Peränen J, Piepponen TP, Saarma M, Heino TI (2009). Evidence that DmMANF is an invertebrate neurotrophic factor supporting dopaminergic neurons. Proc Natl Acad Sci USA.

[16] Hetz C, Chevet E, Oakes SA (2015). Proteostasis control by the unfolded protein response. Nat Cell Biol.

[17] Eesmaa A, Yu LY, Göös H, Nõges K, Kovaleva V, Hellman M, Zimmermann R, Jung M, Permi P, Varjosalo M, Lindholm P, Saarma M (2021). The cytoprotective protein MANF promotes neuronal survival independently from its role as a GRP78 cofactor. J Biol Chem.

[18] De Lorenzo F, Lüningschrör P, Nam J, Pilotto F, Galli E, Lindholm P, von Collenberg CR, Mungwa ST, Jablonka S, Kauder J, Pertri S, Lindholm D, Saxena S, Sendtner M, Saarma M, Voutilainen MH (2020). CDNF rescues motor neurons in three animal models of ALS by targeting ER stress. bioRxiv.

[19] Chhetri G, Liang Y, Shao J, Han D, Yang Y, Hou C, Wang P, Tao X, Shen Y, Jiang T, Feng L, Shen Y (2020). Role of mesencephalic astrocyte-derived neurotrophic factor in alcohol-induced liver injury. Oxid Med Cell Longev.

[20] Danilova T, Galli E, Pakarinen E, Palm E, Lindholm P, Saarma M, Lindahl M (2019). Mesencephalic astrocyte-derived neurotrophic factor (MANF) is highly expressed in mouse tissues with metabolic function. Front Endocrinol (Lausanne).

[21] Glembotski CC, Thuerauf DJ, Huang C, Vekich JA, Gottlieb RA, Doroudgar S (2012). Mesencephalic astrocyte-derived neurotrophic factor protects the heart from ischemic damage and is selectively secreted upon sarco/endoplasmic reticulum calcium depletion. J Biol Chem.

[22] Yan Y, Rato C, Rohland L, Preissler S, Ron D (2019). MANF antagonizes nucleotide exchange by the endoplasmic reticulum chaperone BiP. Nat Commun.

[23] Tronche F, Kellendonk C, Kretz O, Gass P, Anlag K, Orban PC, Bock R, Klein R, Schütz G (1999). Disruption of the glucocorticoid receptor gene in the nervous system results in reduced anxiety. Nat Genet.

[24] Galli E, Rossi J, Neumann T, Andressoo JO, Drinda S, Lindholm P (2019). Mesencephalic astrocyte-derived neurotrophic factor is upregulated with therapeutic fasting in humans and diet fat withdrawal in obese mice. Sci Rep.

[25] Penttinen AM, Parkkinen I, Blom S, Kopra J, Andressoo JO, Pitkänen K, Voutilainen MH, Saarma M, Airavaara M (2018). Implementation of deep neural networks to count dopamine neurons in substantia nigra. Eur J Neurosci.

[26] Bell PA, Dennis EP, Hartley CL, Jackson RM, Porter A, Boot-Handford RP, Pirog KS, Briggs MD (2019). Mesencephalic astrocyte-derived neurotropic factor is an important factor in chondrocyte ER homeostasis. Cell Stress Chaperones.

[27] Danilova T, Belevich I, Li H, Palm E, Jokitalo E, Otonkoski T, Lindahl M (2019). MANF is required for the postnatal expansion and maintenance of pancreatic β-cell mass in mice. Diabetes.

[28] Lindholm P, Peränen J, Andressoo JO, Kalkkinen N, Kokaia Z, Lindvall O, Timmusk T, Saarma M (2008). MANF is widely expressed in mammalian tissues and differently regulated after ischemic and epileptic insults in rodent brain. Mol Cell Neurosci.

[29] Lee A-H, Scapa EF, Cohen DE, Glimcher LH (2008). Regulation of hepatic lipogenesis by the transcription factor XBP1. Science.

[30] Lee AH, Iwakoshi NN, Glimcher LH (2003). XBP-1 regulates a subset of endoplasmic reticulum resident chaperone genes in the unfolded protein response. Mol Cell Biol.

[31] Eletto D, Eletto D, Dersh D, Gidalevitz T, Argon Y (2014). Protein disulfide isomerase A6 controls the decay of IRE1α signaling via disulfide-dependent association. Mol Cell.

[32] Yamamoto K, Sato T, Matsui T, Sato M, Okada T, Yoshida H, Harada A, Mori K (2007). Transcriptional induction of mammalian ER quality control proteins is mediated by single or combined action of ATF6alpha and XBP1. Dev Cell.

[33] Luo SX, Huang EJ (2016). Dopaminergic neurons and brain reward pathways: from neurogenesis to circuit assembly. Am J Pathol.

[34] Burke RE (2003). Postnatal developmental programmed cell death in dopamine neurons. Ann N Y Acad Sci.

[35] Grootjans J, Kaser A, Kaufman RJ, Blumberg RS (2016). The unfolded protein response in immunity and inflammation. Nat Rev Immunol.

[36] Herranen A, Ikäheimo K, Lankinen T, Pakarinen E, Fritzsch B, Saarma M, Lindahl M, Pirvola U (2020). Deficiency of the ER-stress-regulator MANF triggers progressive outer hair cell death and hearing loss. Cell Death Dis.

[37] Neves J, Zhu J, Sousa-Victor P, Konjikusic M, Riley R, Chew S, Qi Y, Jasper H, Lamba DA (2016). Immune modulation by MANF promotes tissue repair and regenerative success in the retina. Science.

[38] Sousa-Victor P, Neves J, Cedron-Craft W, Ventura PB, Liao CY, Riley RR, Soifer I, van Bruggen N, Kolumam GA, Villeda SA, Lamba DA, Jasper H (2019). MANF regulates metabolic and immune homeostasis in ageing and protects against liver damage. Nat Metab.

[39] Montaser H, Patel KA, Balboa D, Ibrahim H, Lithovius V, Näätänen A, Chandra V, Demir K, Acar S, Ben-Omran T, Colclough K, Locke JM, Wakeling M, Lindahl M, Hattersley AT, Saarimäki-Vire J, Otonkoski T (2021). Loss of MANF causes childhood-onset syndromic diabetes due to increased endoplasmic reticulum stress. Diabetes.

[40] Yavarna T, Al-Dewik N, Al-Mureikhi M, Ali R, Al-Mesaifri F, Mahmoud L, Shahbeck N, Lakhani S, AlMulla M, Nawaz Z, Vitazka P, Alkuraya FS, Ben-Omran T (2015). High diagnostic yield of clinical exome sequencing in middle eastern patients with mendelian disorders. Hum Genet.

[41] Rossi AE, Dirksen RT (2006). Sarcoplasmic reticulum: the dynamic calcium governor of muscle. Muscle Nerve.

[42] Maciel L, de Oliveira DF, Mesquita F, Souza H, Oliveira L, Christie MLA, Palhano FL, Campos de Carvalho AC, Nascimento JHM, Foguel D (2021). New cardiomyokine reduces myocardial ischemia/reperfusion injury by PI3K-AKT pathway via a putative KDEL-receptor binding. J Am Heart Assoc.

[43] Airavaara M, Harvey BK, Voutilainen MH, Shen H, Chou J, Lindholm P, Lindahl M, Tuominen RK, Saarma M, Hoffer B, Wang Y (2012). CDNF protects the nigrostriatal dopamine system and promotes recovery after MPTP treatment in mice. Cell Transplant.

[44] Bäck S, Peränen J, Galli E, Pulkkila P, Lonka-Nevalaita L, Tamminen T, Voutilainen MH, Raasmaja A, Saarma M, Männistö PT, Tuominen RK (2013). Gene therapy with AAV2-CDNF provides functional benefits in a rat model of Parkinson's disease. Brain Behav.

[45] Ren X, Zhang T, Gong X, Hu G, Ding W, Wang X (2013). AAV2-mediated striatum delivery of human CDNF prevents the deterioration of midbrain dopamine neurons in a 6-hydroxydopamine induced parkinsonian rat model. Exp Neurol.

[46] Wang L, Wang Z, Xu X, Zhu R, Bi J, Liu W, Wu J, Zhang H, Wu H, Kong W, Yu B, Yu X (2017). Recombinant AAV8-mediated intrastriatal gene delivery of CDNF protects rats against methamphetamine neurotoxicity. Int J Med Sci.

[47] Voutilainen MH, Bäck S, Peränen J, Lindholm P, Raasmaja A, Männistö PT, Saarma M, Tuominen RK (2011). Chronic infusion of CDNF prevents 6-OHDA-induced deficits in a rat model of Parkinson's disease. Exp Neurol.

[48] Hao F, Yang C, Chen SS, Wang YY, Zhou W, Hao Q, Lu T, Hoffer B, Zhao LR, Duan WM, Xu QY (2017). Long-term protective effects of AAV9-mesencephalic astrocyte-derived neurotrophic factor gene transfer in parkinsonian rats. Exp Neurol.

[49] Liu Y, Zhang J, Jiang M, Cai Q, Fang J, Jin L (2018). MANF improves the MPP(+)/MPTP-induced Parkinson's disease via improvement of mitochondrial function and inhibition of oxidative stress. Am J Transl Res.

[50] Chen YC, Baronio D, Semenova S, Abdurakhmanova S, Panula P (2020). Cerebral dopamine neurotrophic factor regulates multiple neuronal subtypes and behavior. J Neurosci.

[51] Chen YC, Sundvik M, Rozov S, Priyadarshini M, Panula P (2012). MANF regulates dopaminergic neuron development in larval zebrafish. Dev Biol.

[52] Mätlik K, Anttila JE, Kuan-Yin T, Smolander OP, Pakarinen E, Lehtonen L, Abo-Ramadan U, Lindholm P, Zheng C, Harvey B, Arumäe U, Lindahl M, Airavaara M (2018). Poststroke delivery of MANF promotes functional recovery in rats. Sci Adv.

[53] Wang Y, Wen W, Li H, Clementino M, Xu H, Xu M, Ma M, Frank J, Luo J (2021). MANF is neuroprotective against ethanol-induced neurodegeneration through ameliorating ER stress. Neurobiol Dis.

